# Supplementary material for: Transcriptional synergy as an emergent property defining cell subpopulation identity enables population shift
Source: Nat Commun. 2018 Jul 3;9:2595. doi: 10.1038/s41467-018-05016-8 (PMC6030214; doi:10.1038/s41467-018-05016-8)
Supplement: Supplementary file 1 — Supplementary Information [file 41467_2018_5016_MOESM1_ESM.pdf]

Supplementary Information

Transcriptional synergy as an emergent property defining cell subpopulation identity enables population shift

Okawa et al., 2018

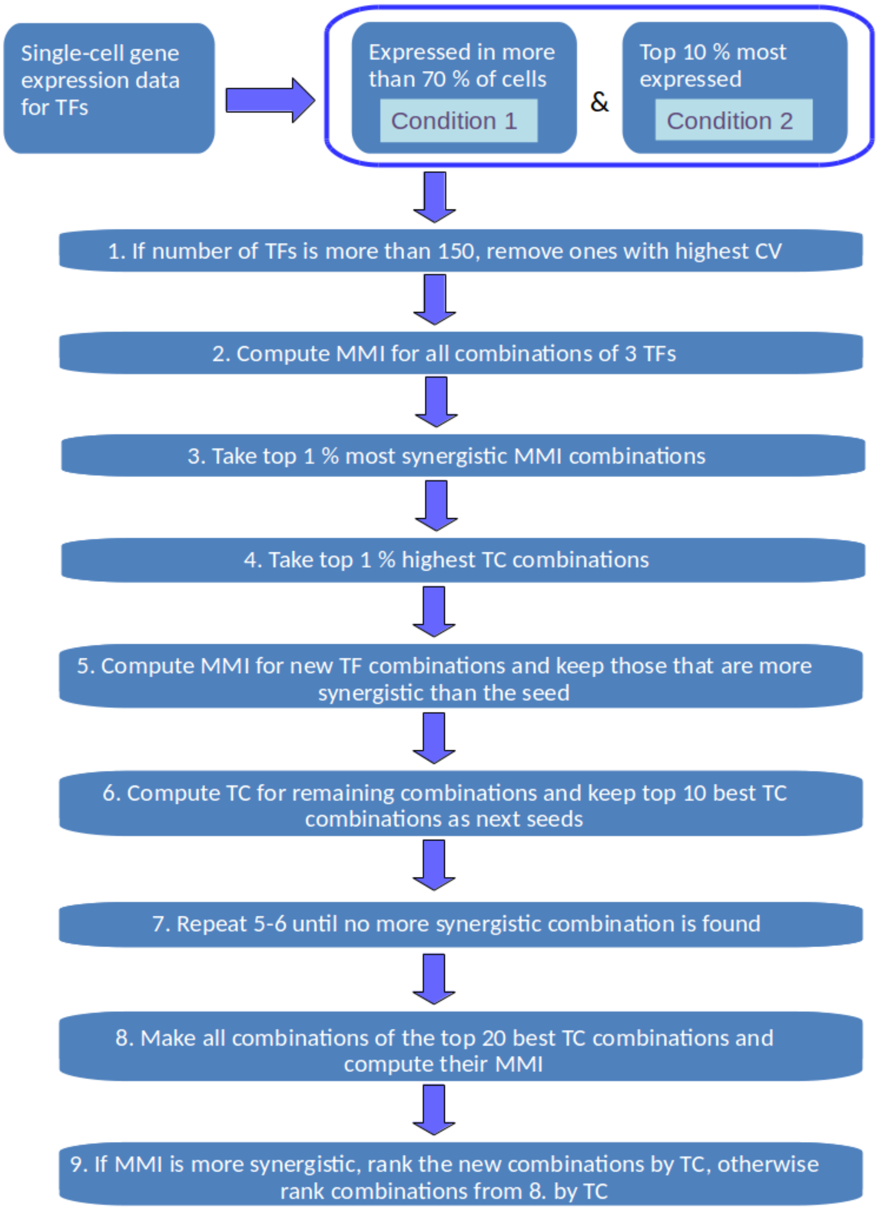

Supplementary Figure 1. Flowchart of the method. First, top 10 % most frequently expressed TFs that are also expressed in more than 70 % of cells within a subpopulation were shortlisted for further analyses. If the number of TFs is more than 150, TFs with highest coefficient of variation were discarded to make the number of TFs <= 150. MMI for all combinations of remaining TFs is computed and top 1 % most synergistic MMI combinations are kept. TC is computed for these remaining combinations and top 1 % highest TC combinations are kept as the initial seed combinations. Then the dynamic heuristic search for synergistic transcriptional cores is applied to the seed combinations (see Figure 2 also). Note, in this study we limited the maximum number of TFs in each core to 15. The search continues until no more synergistic combination is found. Once the search is terminated, MMI for all combinations of the top 3 best TC combinations is computed. If there is more synegistic combination(s) then those combinations are ranked by TC as the final synergistic transcriptional cores.

Supplementary Table 1. Reprogramming factors predicted by Mogrify for cell/tissue types present in both Mogrify and our study.

**Dataset**	**Initial cell type**	**Target cell type**	**Factors**	**PMIDs**
Guo et al. 2015	Bronchial epithelial cell	Lung fibroblast	Foxf2, Sox9, Tbx5, Hoxb5, Foxl1, Foxd1, Nr2f1, Foxc2	
	Endothelial cells	Bronchial epithelial cell	Pitx1, Cdf1, Klf5, Il1b, Adrb2, Esrra, Myc, Hes1	
Gokce et al. 2016	Neurons	NSC	**Sox9**, Zeb1, Cenpf, Zic1, Rfx4, Gli3, **Sox2**, Arnt2	20871603, 23445224
	NSC	Neurons	Cux2, Hes6, Nr2f1, Znf238, Fosb, Usf1, Mafb, Mef2c	
Chu et al. 2016	Many cell types	ESC	**NANOG, SOX2, POU5F1**, TCF7L1, FOXD3, CCNA2	12787504, 16153702, 12787505, 10742100, 10748519
Segerstolpe et al. 2016	Endothelial cells	Mast cells	MYB, **MITF**, GATA1, ELF1, AHR, REL, GFI1, NFATC3	23871207
	Mast cells	Endothelial cells	IL1B, VSX2, HMX1, BHLHE41, CRX, **HES5**, CD86, SIX3	28717251

Their literature evidence as TFs defining cell/tissue types is indicted with PMIDs when available and TFs are in bold.
