# Supplementary material for: Transcriptional synergy as an emergent property defining cell subpopulation identity enables population shift
Source: Nat Commun. 2018 Jul 3;9:2595. doi: 10.1038/s41467-018-05016-8 (PMC6030214; doi:10.1038/s41467-018-05016-8)
Supplement: Supplementary file 3 — Description of Additional Supplementary Files [file 41467_2018_5016_MOESM3_ESM.pdf]

Description of Additional Supplementary Files

File Name: Supplementary Data 1
Description: The 1st column describes datasets and cell subpopulations identified in the original studies. The 2nd column lists one of the top three most synergistic transcriptional cores. TFs are ordered by their gene expression intensity from left to right. TFs in bold have previous experimental evidence as identity TFs.

File Name: Supplementary Data 2
Description: TFs known to maintain cell subpopulation identities were collected from literature evidence.

File Name: Supplementary Data 3
Description: Pair-wise MI was computed among the TFs in each synergistic transcriptional core to see if pair-wise MI could capture the synergistic interaction (see Methods for details).

File Name: Supplementary Data 4
Description: A GRN was inferred for each cell subpopulation using four different metrics and top 10 unique hub TFs were taken as potential candidate TFs that define cell subpopulation identities (see Methods for details).

File Name: Supplementary Data 5
Description: JSD was computed for each TF in each cell subpopulation and top 10 TFs (including ties) were taken as potential candidate TFs that define cell subpopulation identities (see Methods for details).

File Name: Supplementary Data 6
Description: Predicted TFs for converting one cell subpopulation identity into another based on the top three most synergistic transcriptional cores. TFs are ordered from left to right by the mean fold change between the end subpopulation and starting subpopulation. TFs that need to be up-regulated for the conversion are in uppercase letters, whereas those that need to be down-regulated are in lowercase letters.
